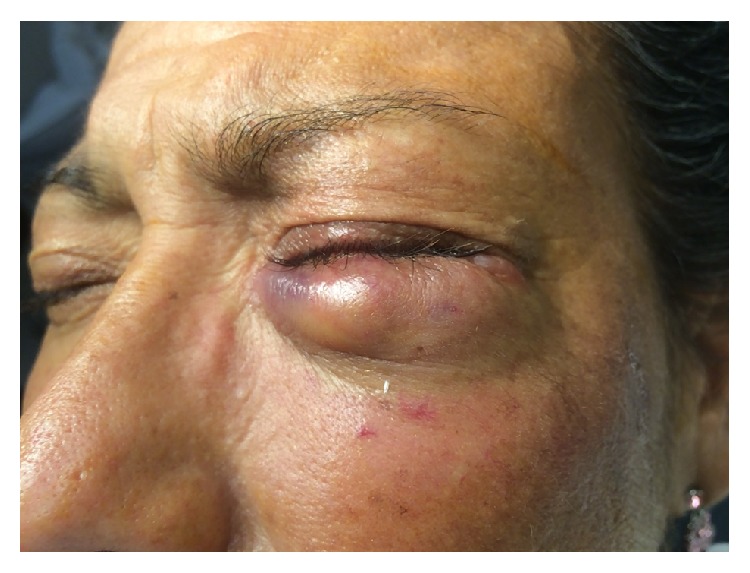# *Escherichia coli* Eyelid Abscess in a Patient with Alcoholic Cirrhosis

**DOI:** 10.1155/2015/827609

**Published:** 2015-09-15

**Authors:** Matthew Stratton, Cara Capitena, Logan Christensen, Miguel Paciuc-Beja

**Affiliations:** ^1^Department of Ophthalmology, University of Colorado School of Medicine, Denver, CO 80045, USA; ^2^Denver Health Medical Center, Denver, CO 80204, USA

## Abstract

*Escherichia coli* (*E. coli*) is a rare cause of ocular infections and has not yet been reported as a cause of an ocular abscess. We describe the case of a 47-year-old woman with a history of alcoholic cirrhosis who presented with painful left lower eyelid swelling that did not improve with oral antibiotics. The abscess was drained and cultures were positive for *E. coli*. Patients with cirrhosis are at increased risk for developing *E. coli* bacterial infections, but to our knowledge this is the first case of an *E. coli* eyelid abscess reported in the literature.

## 1. Introduction

Bacterial infections are frequently seen in cirrhotic patients. Gram-negative organisms, particularly* Escherichia coli *(*E. coli*), are the most commonly identified organisms in such cases.* E. coli *abscesses in patients with cirrhosis have been documented throughout the body, but to our knowledge these infections have never resulted in an eyelid abscess. Eyelid abscesses are typically caused by local skin flora such as* Staphylococcus aureus *(*S. aureus*). We report a case of an* E. coli *eyelid abscess in a 47-year-old woman with alcoholic cirrhosis with previously negative blood and ascites cultures.

## 2. Case Presentation

A 47-year-old woman was referred to the ophthalmology clinic by her primary care doctor for painful swelling of her left lower eyelid that failed to resolve after ten days of oral amoxicillin-clavulanate. Her past medical history was significant for alcoholic cirrhosis confirmed by liver biopsy 2 years prior. Examination of her left eye revealed a 1 cm × 2 cm mobile tender mass within the left lower eyelid (see Figure 1). The remainder of the slit lamp exam was unremarkable. No preauricular lymph node involvement was appreciated. Given the size of the mass and failure to improve on 10 days of antibiotics, the mass was drained via an incision through the palpebral conjunctiva in order to avoid a visible scar. Approximately 1-2 mL of purulent drainage was expressed. Erythromycin ointment was initiated in addition to the oral amoxicillin-clavulanate. Cultures returned positive for* E. coli. *Sensitivities studies showed susceptibility to amoxicillin/clavulanate, ampicillin/sulbactam, cefazolin, cefepime, ceftriaxone, gentamicin, imipenem, levofloxacin, piperacillin/tazobactam, and trimethoprim/sulfamethoxazole. At follow-up two weeks after the initial incision and drainage, the abscess had completely resolved. One month after presentation, however, the abscess recurred and was once again drained, this time via an external approach through the skin. Cultures from the second incision and drainage also grew pan-sensitive* E. coli*. The patient was advised to follow up with her primary care doctor for evaluation of possible bacteremia. Unfortunately blood cultures were never taken and the patient was lost to follow-up. Of note, the patient had undergone blood and ascites fluid cultures several months prior to developing these eyelid abscesses, both of which grew no organisms at that time.

## 3. Discussion

Patients with cirrhosis have a significantly higher incidence of bacterial infections than the general population. Among hospitalized patients, these infections are estimated to be 5–7x more likely in cirrhotic patients [1]. There are several proposed mechanisms for this increased risk. First, cirrhosis culminates in multiple immune deficiencies. In one study, bactericidal function of IgM for certain strains of* E. coli *was impaired in 80% of patients with cirrhosis. Additionally, 60% of patients with alcoholic liver disease were found to have decreased chemoattractant activity and ability to mobilize polymorphonuclear cells [2]. Cirrhotic patients may also suffer from complement deficiencies, impaired opsonization of pathogenic organisms, and a decreased number and function of Kupffer cells in the liver itself [2]. Portal hypertension, often seen in cirrhotic patients, can lead to increased permeability of the gut wall, cytokine dysfunction, reduced small bowel motility, and bacterial overgrowth in the gut, all of which predispose individuals to transmural migration of enteric bacteria. Additionally, chronic alcohol ingestion is known to cause disintegrity of the gut mucosa leaving an individual at further risk of transmural migration of enteric organisms into the circulation [3, 4]. Given these immune and gastroenterologic changes, cirrhosis is often considered to be a form of acquired immunodeficiency, explaining the high incidence of bacteremia documented in patients with the disease [5].

Prospective analysis of bacterial infections in patients with cirrhosis has demonstrated that while bacterial peritonitis is the most common infection, soft tissue and skin infections are also quite common and represent approximately 11–33% of infections in cirrhotic patients [6, 7]. Gram-negative bacteria, in particular* E. coli*, are the most common causative organism in bacterial infections in cirrhotic patients, regardless of the location of the infection [8, 9]. Gram-positive local skin flora are traditionally the organisms responsible for eyelid infections, with* S. aureus *being the most common etiology [10].* E. coli *has been implicated in a number of ocular infections such as endophthalmitis and corneal ulcers, but, to date, there have been no reports of* E. coli *cultured from an eyelid abscess [11, 12].

While there are no blood cultures to confirm bacteremia in this patient at time of infection, we theorize that the patient's relative immunosuppression and altered gastroenterological environment, both secondary to her cirrhosis, allowed translocation of* E. coli *from the GI tract ultimately resulting in an eyelid abscess. The location of this* E. coli *infection is unique and unreported in the literature. However, the link between cirrhosis and* E. coli *infections in cirrhotic patients is well established, as is the pathophysiologic mechanism by which these enteric organisms may have spread throughout the body.

## Figures and Tables

**Figure 1:**